# Slow Heat-Based Hybrid Simulated Annealing Algorithm in Vehicular Ad Hoc Network

**DOI:** 10.1155/2023/9918748

**Published:** 2023-02-17

**Authors:** Pavan Kumar Pagadala, P. Lalitha Surya Kumari, Deepak Thakur, Vivek Bhardwaj, Mohammad Shahid, Abdulrajak Buradi, Abdul Razak, Abiot Ketema

**Affiliations:** ^1^Computer Science and Engineering, Koneru Lakshmaiah Educational Foundation, Hyderabad, Telangana, India; ^2^Chitkara University Institute of Engineering and Technology, Chitkara University, Punjab, India; ^3^School of Computer Science and Engineering, Lovely Professional University, Jalandhar 144411, India; ^4^Department of Electrical Engineering, Galgotias College of Engineering and Technology, 1, Knowledge Park, Phase II, Noida 201306, UP, India; ^5^Department of Mechanical Engineering, Nitte Meenakshi Institute of Technology, Bangalore, India; ^6^Department of Mechanical Engineering, P. A. College of Engineering (Affiliated to Visvesvaraya Technological University, Belagavi), Mangaluru 574153, India; ^7^Department of Biosystems Engineering, Institute of Technology, Hawassa University, Hawassa, Ethiopia

## Abstract

Vehicular ad hoc networks (VANETs) using reliable protocols of routing have become crucial in identifying the changes to topology on a continuous basis for a large collection of vehicles. For this purpose, it becomes important to identify an optimal configuration of these protocols. There are several possible configurations that have been preventing the configuration of efficient protocols that do not make use of automatic and intelligent design tools. It can further motivate using the techniques of metaheuristics like the tools, which are well-suited to be able to solve these problems. The glowworm swarm optimization (GSO), simulated annealing (SA), and slow heat-based SA-GSO algorithms have been proposed in this work. The SA is a method of optimization, which imitates the manner in which the thermal system has been frozen down to its lowest state of energy. In the GSO, there is guidance to the rules of feasibility, where the swarm converges to its feasible regions very fast. Additionally, for overcoming any premature convergence, there is a local search strategy that is based on the SA and is used for making a search that is near to its true optimum solutions. Finally, this sluggish temperature-based SA-GSO algorithm will be employed to solve routing problems and problems of heat transfer. There is a hybrid slow heat SA-GSO algorithm with a faster speed of convergence and higher precision of computation that is more effective in solving problems of constrained engineering.

## 1. Introduction

The vehicular ad hoc networks (VANETs) refer to the self-configuring networks that contain a large collection of vehicles or elements in the roadside infrastructure and are connected to one another without the need to have the underlying infrastructure. Recently, technologies based on WiFi (IEEE 802.11-based) were employed to deploy these networks. The limitations of coverage occur as the network topology changes due to the elevated mobility of the vehicles. For the same reasons, without the need for an entity such as a central manager, the task of routing can be very challenging. Thus, the provision of an efficient strategy for routing is important in deploying the VANETs for increasing the quality of service (QoS) to the extent possible [1].

The VANET includes vehicles travelling in wider areas at speeds that are high enough to cause a rapid change in topology that can present a challenge to the reliable and efficient dissemination of messages. There are some more special features of the VANET: the nonuniform distribution of nodes, the lack of an administrative entity that is centralized, the topology of a fragile link, the dynamic changes to node density, the large-scale real-time delay, which is stringent, and the impact of the behaviour of drivers on the topology of the network. These features can make it very challenging to give suitable QoS service to the VANET, which is a key routing technology [2].

The VANET has three primary components: the On_Board_Units (OBU) installed in vehicles, the Road_Side_Units (RSU) installed on the roads, and wired (inter-RSU) or wireless (the OBUs to the RSU) channels. These vehicles will correspond with one another through the OBU for the exchange of traffic and information on infotainment through broadcasting their messages [3]. The routing architecture in the VANET is primarily similar to the routing architecture in networks that are connectionless. In the same way, there is a conceptual terminology for the VANET that has been elaborated vastly compared to its peers that are roughly equivalent. The routing architecture of the VANET will employ hop-by-hop connectionless open routing systems in [4]:

- A new set of protocols of routing that permit the end systems and the intermediate systems in order to gather and further distribute information for determining routes.
- A base for routing information will also include information from which the routes that are between all end systems are computed based on the directory information or the routing information, which is a concept and will not exist as one single entity. The routing information base is considered as a combined (or distributed) information of the whole subsystem that is connected to routing and its relevant connectivity, which is found between the subsystem and its components.
- This routing algorithm employs the information contained within routing information based for getting routes among the end systems.

In the case of wired networks, the QoS will be defined using two different terms: the delay and the throughput. In the case of the VANETs, the QoS is challenging to define and can be satisfied owing to several features, like the changing VANET network topology, the scalability, the deployment of heterogeneous environments, the high density, the mobility of nodes, and the broadcasting that is delay constrained. In the VANET network, data transmitted can be real-time data like traffic messages and the streaming of both audio and video like meteorological information and e-maps. This imposes several needs for performance and maintaining the QoS for the developers of the VANETs [5]. There can be packet delay, collision, or congestion on the increase owing to the exploding of several vehicles that compete for a common allocation of the wireless medium. Both transmitting and receiving vital data within a fixed time are very critical for this network [6].

Owing to its perspective of a high level, the mobile ad hoc networks (MANET) may not always be well-suited for the VANETs. There are various other methods for tuning the parameters of routing that were introduced in order to make sure there was a high-performance level that met the specific traits of the VANETs. There are many other methods that tune the parameters of routing introduced for ensuring a high performance in its dynamic network. Most of these are considered to be the QoS to be a major need for enhancing the efficiency of routing and overcoming various problems like node density that is unpredictable. Furthermore, in order to address the traits of these dynamic scenarios, the investigation may be extended further by various areas of research, like the optimization-based multiple objectives that enhance delivery through device-to-device (D2D) communications. Most of such recent investigations were taken to be the QoS for optimizing routing and its efficiency by means of introducing the concept of optimization with techniques of particle swarm optimization (PSO) to bring down the road constraints on the performance of routing [7].

Several applications of the VANET are dependent on the protocols of routing. So, there are some more strategies for optimal routing to ensure better resource utilization for the purpose of finding configurations or parameters of the current protocols of the MANET that will be able to improve performance, which makes a difference between the network and whether or not it works. This means networks work with a high level of routing suffering from congestion to make sure there is a dependable message delivery [8]. For the purpose of this proposed work methodology, a hybrid slow heat SA-GSO is used for the VANET routing. The respite of the paper is ordered as follows: Section 2 has discussed a few works in the literature. The various techniques used and the proposed method are detailed in Section 3. The results of the experiments were discussed in Section 4 and Section 5 concludes the work.

## 2. Related Works

Pagadala and Saravana Kumar [9] proposed a narrative setting up algorithm used for reliable routing. This structure and classification were able to distribute the workload on alternative routes to avoid congestion and bring down the cost of travel. This will further ensure any reliable routing with a bandwidth need that was low and manages the packets of large size thus dividing them into sections and transmitting them through alternative paths. Thus, the algorithm for routing was obtained, and this was subject to several constraints of the QoS used for secure VANET communication.

In order to maximize the capacity of transmission and keep the QoS at an application level in terms of safety, there was a scheme of optimization that was introduced by Shaikh and Hingoliwala [10] to adjust the parameters of transmission, and these are employed in the real test beds.

Zhao et al. [11] proposed the bees life algorithm (BLA) based on the bee colony optimization (BCO) algorithm, which was utilized in solving the problems of QoS-multicast routing (QoS-MRP) in the VANETs, which was an NP-Complete problem having multiple constraints. It further followed two different behaviours in their nature, and the BLA was thus applied to solving the QoS-MRP using four different objectives: cost, jitter, delay, and bandwidth. This had further been submitted under three different constraints: the maximum permissible delay, the maximum allowed jitter, and the minimum requested bandwidth.

Bitam and Mellouk [12] proposed a Cat_Swarm_Optimization (CSO)-based Geographic_Routing (GR) procedure called the CSO_GR. There was yet another fitness function, which was used for optimizing the effect of parameters on the choice of the subsequent forwarding vehicle. There was some significant improvement observed in the performance of this protocol, which was assessed through simulation. There was a significant level of improvement that was observed in the performance in connection to the normalized load of routing and the packet delivery ratio.

Kasana and Kumar [13] introduced another QoS_Aware_Routing in VANETs (QARV) where the packets had reached a destination at the time of satisfying their QoS. This was a new concept known as the terminal intersection and was used for reducing congestion and the time taken for route exploration. For the purpose of this work, the ACO and the BCO were employed to achieve results. The work further provided a comparative analysis of the parameters of the performance of the algorithms.

Kaur et al. [14] proposed another improved jumbled Frog_Leaping Algorithm_Based QoS-controlled multicast routing (ISFLABMR) to estimate an optimal multicast tree in VANETs. The proposed method minimized the transmission cost to 22%, and this was done by decreasing the multicast cluster formation at the time of multicasting by using both limited and universal optimizations. The mock-up of the proposed method showed a major rate of about 24% and 21% of middling packet latency, along with the consumption of energy that was incurred during multicast routing.

Malathi and [15] made an evaluation of the Optimized_Link_State_Routing Protocol (OLSR) in order to improve performance. The OLSR protocol performed well in networks with frequent changes in node topology. This was done by means of defining the problem of optimization wherein hybridization of the metaheuristics had been defined. This work further contributed to the idea of a combination of both the GA and the SA (hybrid GA-SA) for enhancing the recital of the technique of individual search for the predicament of optimization [16]. Simulation results showed that the tuned configurations of the OLSR and their results were comparatively consistent because of their better efficiency in QoS and communication compared to the standard, which makes them fit to be utilized in the configurations of the VANET. The comparison of GA, ACO, PSO, and GSO algorithms in terms of their benefits and drawbacks is provided in Table 1.

## 3. Methodology

The VANETs have major challenges to face, like fast changes to topology, a link lifetime that is low, or a large number of vehicles participating at a given time. The first two issues encouraged researchers to be able to propose the protocols of geographical routing for the VANETs making routing decisions on local information without the necessity for the construction of construct end-to-end routes [18].

The protocols of geographical routing have evolved from taking into consideration the geographical distance among nodes based on their forwarding criteria, which included some additional metrics such as direction and speed. The routing judgment for the next upcoming hop will be dependent on the subsequent forwarding hop, which was the best based on a particular parameter or metric. The option of the preeminent node can maintain its predominance in the criterion of routing among the other protocols of geographical routing. At the same time, there can be a hop-by-hop strategy of forwarding for the VANETs through the application of a family heuristic known as the “local search algorithms.” For the purpose of this work, this hybrid SA-GSO with the hybrid slow heat SA-GSO algorithm had been designed for improving results obtained using algorithms of local search in the discrete problems of optimization.

### 3.1. Simulated Annealing (SA)

The adaptation of SA to the protocols of VANET routing attempts to provide randomness to the process. This helps decrease the time a packet is able to approach its destination. The SA employs equation (1) for this purpose. In the event that there are neighbours that are closely related based on their contemporary node (the forwarding phase), the equation gives the probability of choosing any random node to be the forwarding hop. If not, at the time of recovery, when there is no closer node, equation (1) will be the probability of choosing a node, which is farther away compared to the current node. The primary idea was to choose a worse node compared to the current one based on the distance to avoid buffering and to take into consideration forwarding nodes [19].

(1) $$\begin{array}{c} p=e^{-\gamma \left(\left|d_{s-s^{\prime }}\right|/d_{s}\right)}\text{.} \end{array}$$

**Figure 1:**
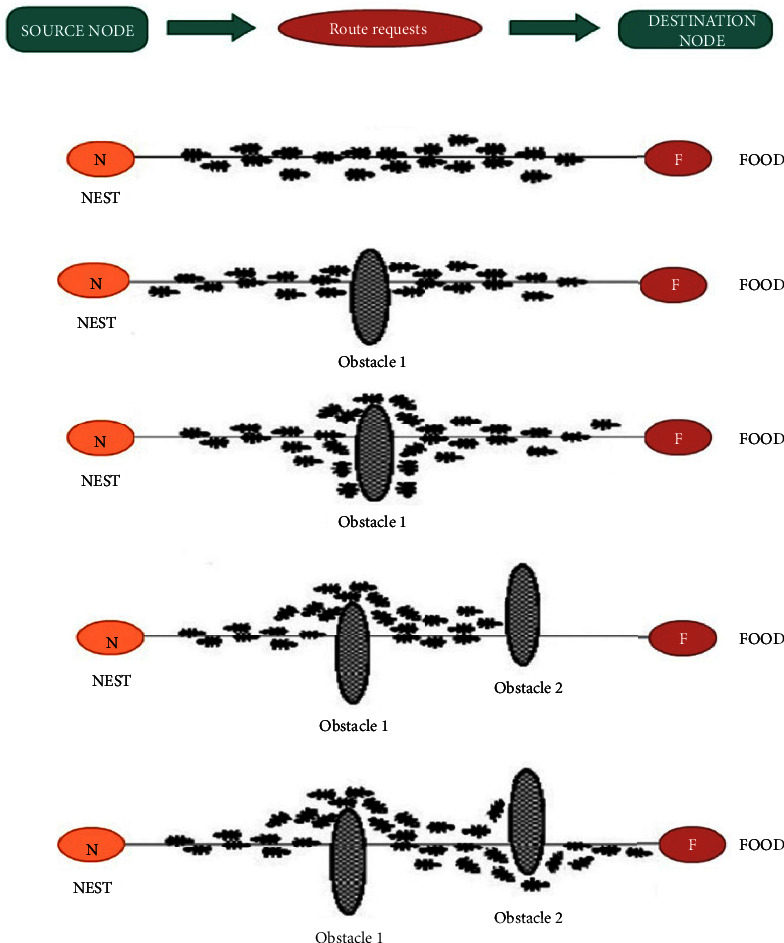
GSO route to resources.

These metrics for computing probability *p* in equation (1) are as follows:

- The tangible distance is from the progress node to the objective node *d*_*s*_. When the data is transmitted, it approaches the destination, and the *d*_s_ will thus decrease. This can bring down probability *p*.
- |*d*_*s*−*s*′_| denotes the absolute value for the distances and their differences on their best forwarding contestant duly to its destination (called *d*_*s*′_) and also its current node to the destination *d*_s_. In case the *d*_*s*−*s*′_ is found to be high, it contributes to the reduction of probability *p*. The primary idea of such a numerator was to ensure that the subsequent position of the packet was by neatly choosing the one which was the closest and not very far-flung from its in progress point (i.e., the contestant that is the best will improve in distance), and this may be a good option to make a random choice and attempt other paths that were not explored [19,20] otherwise owing to the benefit of its greedy selection that denotes the selection of its best candidate (Figure 1).
- *γ* denotes the parameter of tuning to balance the behaviour of randomness. In case it is near zero, *p* will be one. Conversely, if *γ*⟶*∞*, then *p*⟶0.

The root equation for finding the nearest neighbours is as follows:

(2) $$\begin{array}{c} \overset{n=1}{\underset{k=1}{\sum }}k=\left(\frac{n*\left(n-1\right)}{2}\right)\text{.} \end{array}$$

### 3.2. Glowworm Swarm Optimization (GWO) Algorithm

In GSO, the methodology will be as follows: every glowworm tends to allocate space to its objective function. They carry their luciferin and are within their respective scope of vision within their range of local decisions. The intense brightness of the glowworms is found in the location of their objective function and utility value. The position is better if the glow is brighter [17]. The glowworms will search in their neighbour set for a new local range of decisions. Furthermore, the size of its local-decision range can be influenced by the number of neighbours. The radius is decided by the density of the glowworms in the neighbourhood; if the density is less, the radius is increased; otherwise, it is reduced [16].

Every glowworm *i* will encode its objective function value, which is *J*(*x*_*i*_(*t*)) and this will be stayed at its present position *x*_*i*_(*t*) within a new luciferin value *L*_*i*_, which will be broadcasted in its neighbourhood. There is a group of neighbours *N*_*i*_(*t*) of glowworm *i* that contains glowworms with a higher luciferin value in the neighbourhood, and this is updated based on the formula (7) for every iteration.

The update of a local-decision radius is as follows (3):

(3) $$\begin{array}{c} r_{d}^{i}\left(t+1\right)=\min\;\left\{r_{s},\max\;\left\{0,r_{d}^{i}\left(t\right)+\beta \left(n_{t}-\left|N_{i}\left(t\right)\right|\right)\right\}\right\}\text{.} \end{array}$$

The *r*_*d*_^*i*^(*t*+1) denotes the glowworm *i*'s range of local-decision, which is *t* + 1 iteration; *r*_*s*_ denotes node range; *n*_*t*_ denotes the threshold of the neighbourhood; and finally, parameter *β* will affect the actual rate of change in the range of neighbourhood. The actual number of glow found in a local-decision range is as follows (4):

(4) $$\begin{array}{c} N_{i}\left(t\right)=\left\{j:\left\|x_{j}\left(t\right)-x_{i}\left(t\right)\right\|<r_{d}^{i};l_{i}\left(t\right)<l_{j}\left(t\right)\right\}\text{.} \end{array}$$

*x*_*j*_(*t*) denotes the position of the glowworm *i*, wherein the position is at *t* iteration. *l*_*j*_(*t*) denotes glowworm *i* ‘s luciferin at iteration *t*. Every glowworm *i* will choose a new neighbour *j* having probability *p*_*ij*_ (*t*) and will duly move towards it. The movements have been based on the local information that enables the glowworms to divide into certain disjoint subgroups exhibiting a taxis-behaviour towards the colocation at the optima of the objective function [21].

There is a probability distribution for selecting the neighbour as follows (4):

(5) $$\begin{array}{c} p_{ij}\left(t\right)=\frac{l_{j}\left(t\right)-l_{i}\left(t\right)}{\underset{k\in N_{i}\left(t\right)}{\sum }l_{k}\left(t\right)-l_{i}\left(t\right)}\text{.} \end{array}$$

The movement update is as follows (6):

(6) $$\begin{array}{c} x_{i}\left(t+1\right)=x_{i}\left(t\right)+s\left(\frac{x_{j}\left(t\right)-x_{i}\left(t\right)}{\left\|x_{j}\left(t\right)-x_{i}\left(t\right)\right\|}\right)\text{.} \end{array}$$

The luciferin update is as follows (7):

(7) $$\begin{array}{c} l_{i}\left(t\right)=\left(1-\rho \right)l_{i}\left(t-1\right)+\gamma J\left(x_{i}\left(t\right)\right)\text{.} \end{array}$$

The *l*_*i*_(*t*) denotes a luciferin value for the glowworm *i* at iteration *t*, and *ρ* ∈(0,1) will result in showing the cumulative effectiveness for the path that is followed by glowworms within their present luciferin values, parameter *γ* will scale the values of fitness functions and *J*(*x*_*i*_(*t*)) denotes the test function value.

Every glowworm *i* chooses a neighbour *j* with probability *p*_*ij*_(*t*) and moves towards it. The moves are duly based on their local information and this can allow the glowworms to divide themselves into disjoint smaller group exhibiting a taxis-behaviour towards and this will ultimately colocate its multiple optima for the objective function given.

### 3.3. Hybrid SA-GSO Algorithm

The SA [22] is a proven algorithm of global optimization owing to its advantage, which are described as follows: (1) the suitability of the problem in a wider area; (2) not having any restriction in the form of any cost function; (3) having a high probability of finding global optimization; and (4) using programming for easy functional implementation. The *S*_*A* is not a universal one and its efficient performance is dependent on four of the “enough;” (1) a high enough initial temperature, (2) a slow enough cooling of temperature, (3) an often enough sampling of parameter, space and (4) a low enough stop temperature. All these needs will make it converge in a slow manner.

This work further proposed a new optimization algorithm that followed the GSO and the SA with the traits of the SA and the GSO. Both algorithms were mixed and iterated within the calculation. By using the criterion of Metropolis in the SA, there were some glowworms that reached a particular level in terms of similarity. There was another new solution that was generated by the random disturbance of the SA for reaching a new location that can increase the glowworm and its diversity to steer clear of the triumph spellbound in local best possible. For the SA-GSO, GSO of fast convergence and a global convergence for the SA algorithms, there was a “premature” convergence of the GSO that was overcome and convergence speed improved by a combination of this two-algorithm search [23].

A hybrid SA-GSO is its basic framework, and this will update an optimum population position by employing the strategy based on the rules of feasibility found in the process of search [24]. It also adopts a strategy of local search using SA for finding the optimal position for every generation [24]. Additionally, initial warmth is computed with the pragmatic formula (8):

(8) $$\begin{array}{c} T\left(0\right)=\frac{f_{\max}-f_{\min}}{\ln\left(0,1\right)}\text{,} \end{array}$$

where both *f*_max_ and *f*_min_ denote the maximum and the minimum objective values for solutions in their initial swarms [25]. Aside from this, there is exponential annealing, which is *T* (*k* + 1) = *λT* (k), and the rate of annealing will satisfy 0 < *λ* < 1. This procedure of the hybrid SA-GSO is as follows:

1. There is a random initialization of the positions of the N individuals within the search space, and the initial temperature *T* (*k*) is calculated in accordance with equation (8). Now initialize an optimal position *X*^*∗*^ with an optimal value *f* (*X*^*∗*^) for the population based on the rules of feasibility.
2. The whole population will be divided into two random swarms: a glowworm swarm and a simulated annealing swarm.
3. The GSO is implemented for glowworm swarm, and in accordance with the rules of feasibility, determine its current optimal position, which is *X*_GSO−best_^*k*^ of glowworm swam_1_, and apply a strategy of local search, which is based on the SA to the *X*_GSO−best_^*k*^ and finally get its new position *X*_GSO−newbest_^*k*^ [26].
4. Now implement the SA swarm, and based on the rules of feasibility, determine its current optimal position, which is *X*_SA−best_^*k*^ of its SA swam_2_, and apply a strategy of local search that is based on the SA to *X*_SA−best_^*k*^ and obtain a new position *X*_SA−newbest_^*k*^.
5. Based on the rules of feasibility, if the *X*_GSO−newbest_^*k*^ is found to be better than the *X*_SA−newbest_^*k*^, then *X*_GSO−newbest_^*k*^ will update *X*_SA−newbest_^*k*^, and if not, *X*_SA−newbest_^*k*^ will update *X*_GSO−newbest_^*k*^. Additionally, based on the rules of feasibility, the optimal position X∗ is updated, and the optimal value *f* (*X*^*∗*^) for the whole population is obtained.
6. In case maximum iterations are arrived at, then stop with the optimal output position *X*^*∗*^ and an optimal value *f* (*X*^*∗*^) for the whole population; if not, let *T* (*K* + 1) = *λT* (k), *k* = *k* + 1, and move back to Step 3.

### 3.4. Proposed Hybrid Slow Heat-SA-GSO Algorithm

**Figure 2:**
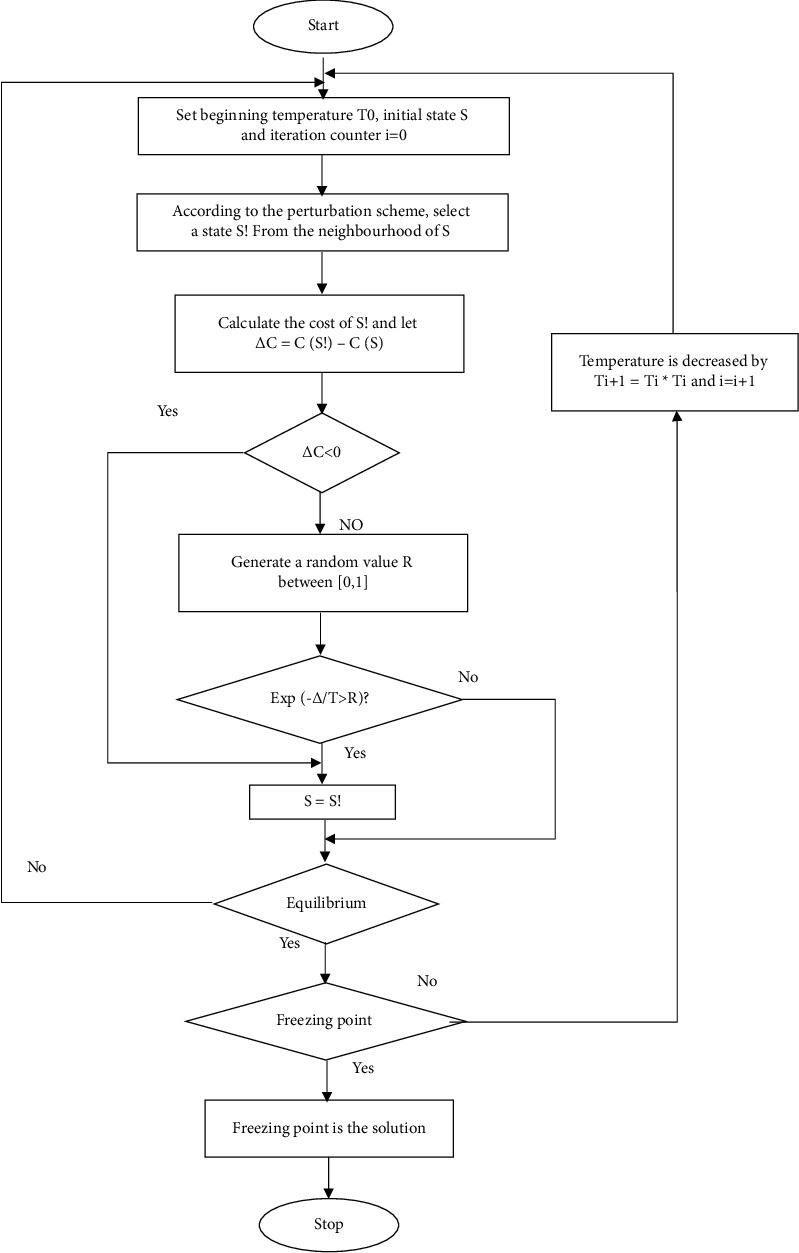
Flow chart of slow heat-based hybrid SA-GSO algorithm.

Figure 2 shows the flow chart slow heat-based hybrid SA-GSO algorithm. Employing the SA in connection to the construction design, which is used for a geometrical optimization of the cavities, is not duly investigated, but it represents novel and important work. After this, there is an investigation of a suitable annealing and cooling schedule, which is a very important subject [27]. Applying the algorithm without any adjusted configurations may result in errors in obtaining the global optimal shapes and the influence of every parameter (degree of freedom (DOF)) that is over the performance of its thermal performance. Generally, it may not be easy to compare them with the ones achieving an exhaustive search (ES). This way, the work has the intention of reaching a commendation on the most excellent schedules of cooling up for a dependable optimization along with the SA metaheuristic employed for the optimization of the multifarious cavities within the construct framework of design [28].

It is assumed that a *Y*-shaped cavity can act to remove heat that is generated using a solid domain (the grey region). This will seek the most favourable geometry for the hollow space that is given by the variation of degrees of free will, which are: the *H*/*L*, *t*_1_/*t*_0_, *L*_1_/*L*_0_, and *α*. The temperature field, which is within a solid domain, has been solved for each of the study cases and its maximum temperature. An ideal thermal performance for this system was obtained at the time the maximum temperature, which was within the solid domain, was brought down.

The primary objective was to play down all dimensionless and highest overindulgence warmth (*θ*_max_) found within the unyielding sphere of influence by a following dimensionless equation (9):

(9) $$\begin{array}{c} \theta_{\max}=\frac{T_{\max}-T_{\min}}{q^{m}\left(A/K\right)}\text{.} \end{array}$$

Based on Section 3.3, the hybrid SA-GSO is used for the slow heat process, and the fast annealing method is used for the cooling schedule [21] with a decrease that is faster (9):

(10) $$\begin{array}{c} T_{k}=\frac{T_{0}}{\left(k+1\right)},k=1,\cdots ,\infty \text{.} \end{array}$$

This can decrease sharper than the *T*_*k*_ = (*T*_0_/ln (*k* + *c*)), *k* = 1, ⋯, *∞*. But it will have to match the temperature decrease compared to the solution of the neighbourhood and its process of generation. Saravana Kumar et al. [29] divided the heat values from [0, *T*_0_] into *K* interval gaps by finding the Temp_*k*_, *k* = 1,…, *K*. This fast method of simulated reannealing had been presented by Ingber in the year 1989 by Ingber. It has a cooling schedule which is as follows (10):

(11) $$\begin{array}{c} T_{k}=T_{0}\exp\;\left(-ck^{\left(1/n\right)}\right),k=1,\cdots ,\infty \text{,} \end{array}$$

where *c* denotes the dimension factor. Ingber [30] had also second-hand another slower programme of (9) that is shown in (11):

(12) $$\begin{array}{c} T_{k}=\frac{T_{0}}{{\left(k+1\right)}^{\left(1/n\right)}},k=1,\cdots ,\infty \text{.} \end{array}$$

Even though there are several cooling schedules mentioned, there is a scheme of geometric cooling as in equation (13) that was anticipated by [31].

(13) $$\begin{array}{c} T_{k+1}=\alpha T_{k},k=0,\cdots ,\infty \text{,} \end{array}$$

where *α* ∈ (0, 1) denotes a constant and is used widely as a popular cooling schedule since it compromises the CPU time and is quality [32–34].

## 4. Results and Discussion

**Figure 3:**
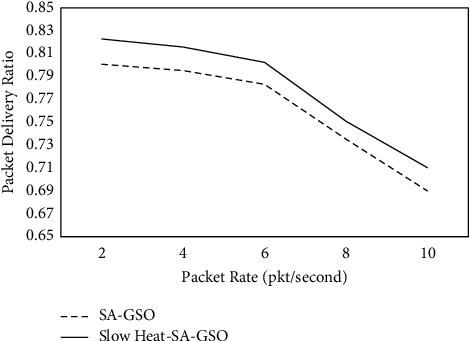
Packet_delivery_ratio for slow heat-SA-GSO.

The proposed SA-GSO and slow heat-SA-GSO methods are evaluated. The experiments are carried out using a ratio of 2 to 10 packet rates and everything in between. The proposed methods are evaluated for metrics like average delay, packet delivery ratio, and a middling number of hops.  Table 2 and Figure 3 depict the results achieved for packet delivery ratio.

**Figure 4:**
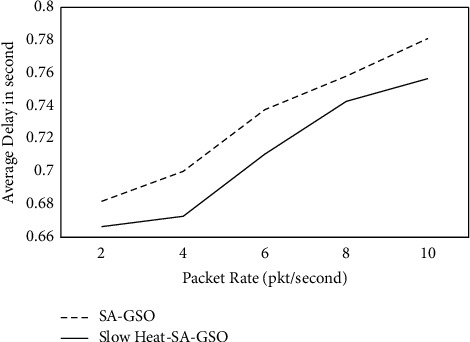
Average_delay in second for slow heat-SA-GSO.

It can be observed from Table 2 and Figure 3 that the slow heat-SA-GSO has a higher PDR in the range of 2.11% to 2.89% than SA-GSO.  Table 3 and Figure 4 show the results for the average delay seen for SA-GSO and slow heat-SA-GSO.

**Figure 5:**
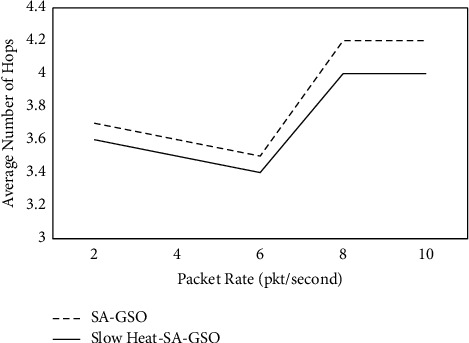
Average_number_of_hops for slow heat-SA-GSO.

It is observed from Table 3 and Figure 4 that the proposed slow heat-SA-GSO has a lower average delay in the range of 2.05% to 3.99% when compared with SA-GSO. The average delay was computed in seconds.  Table 4 and Figure 5 depict the average number of hops for slow heat-SA-GSO.

From Table 4 and Figure 5, it is observed that the proposed slow heat-SA-GSO has a higher average number of hops when compared with SA-GSO [35, 36].

## 5. Conclusion

For the VANETs, there are limitations in terms of capacity and coverage of channel of the WiFi technologies, aside from the high node mobility and various factors that lead to packet losses, changes in topology, and fragmentation of the network. In the case of an optimal strategy of routing to utilize the resources, there is a possibility to deploy an efficient VANET. The routing data packets in the VANETs can be a demanding task as there is no other central administrator entity that is in incriminate of identifying the available routes of routing surrounded by these nodes. So, there may be a great deal of such efforts that are dedicated to designing some of these efficient protocols of routing. For the purpose of this work, a hybrid slow heat-based SA-GSO algorithm was proposed. Every GSO iteration contained a phase of luciferin update, which was followed by the progress phase that was based on a rule of transition. This was a metaheuristic that was used widely in both optimization and combinatorial problems. The algorithm also has a constraint on the cooling schedule (the drop-off of its warmth) that is defined for every problem in an empirical manner. For the purpose of this work, the aim was to identify the most outstanding cooling schedule (*s*) in geometric optimization for a problem of heat transfer. The GSO was integrated using slow heat, aside from the technology of constriction dispensation, which is based on the rules of feasibility that were used for updating an optimum population position. In order to escape a local optimum, the strategy of local search that was based on the SA was applied to its best solution for every generation. The results proved the slow heat-SA-GSO to have a higher rate of PDR by about 2.72% for the 2 data packet rate, by about 2.57% for the 4 data packet rate, by about 2.43% for the 6 data packet rate, by about 2.11% for the 8 data packet rate, and finally by about 2.89% for the 10 data packet rate on being compared to the SA-GSO, respectively.

## Figures and Tables

**Table 1:** Comparison of GA, ACO, PSO, and GSO algorithms [17].

Algorithm				
Items	GA	ACO	PSO	GSO
Year	1975	1992	1995	2005
Author	John Holland	Marco Dorigo	James Kennedy and Russell Eberhart	K.N. Krishnanand and Debasish Ghose
Optimization	Discrete optimization	Meta-heuristic optimization	Stochastic optimization	Meta-heuristic optimization
Parameters	Reproduction, crossover, mutation	Construct ant solutions, daemon actions (optional), and update pheromones	Current velocity, personal best, neighborhood best	Initialization, updating luciferin, movement, updating the local- decision range
Purpose	Find the best among others	Find the shortest path	Reach target with minimal duration	Find the local finest solution
Advantages	(1) Efficient means of investigating large combinatorial problems and can solve them (2) Many orders of magnitude faster than exhaustive „brute force‟ searches	(1) Inherent parallelism (2) Positive feedback accounts for rapid discovery of good solutions (3) Efficient for traveling salesman problem and similar problems (4) can be used in dynamic application (adapts to changes such as new distances, etc)	(1) PSO can be applied into both scientific research and engineering use (2) It has no overlapping and mutation calculation (3) The search can be carried out by the speed of the particle (4) PSO adopts the real number code, and it is decided directly by the solution	(1) GSO can deal with highly nonlinear, multimodal optimization problems naturally and efficiently (2) GSO does not use velocities, and there is no problem as that associated with velocity in PSO (3) The speed of convergence of GSO is very high in probability of finding the global optimized answer
Disadvantages	(1) Computationally expensive (2) Some problems require many days or weeks to run (3) However often still faster than force (4) Blind, to direct a GA towards optimal solution area if know	(1) Theoretical analysis is difficult (2) Sequences of random decisions (not independent) (3) Probability distribution changes by iteration (4) Research is experimental rather than theoretical	(1) Tendency to a fast and premature convergence in midoptimum points (2) The method cannot work out the problems of scattering and optimization (3) Slow convergence in refined search step	(1) GSO is poor in high dimensional problems (2) In GSO, the dynamic change of decision domains in the method of glowworms moving, the algorithm slows convention speed and has poor local search ability delayed in the iteration
Medical field	Genetic algorithm outperformed optimizes the artificial neural networks among others	ACO also optimizes the artificial neural networks for applications in medical image processing	(1) Detection of brain tumor using image segmentation (MRI) (2) PSO used for optimize the artificial neural networks for applications in medical image processing	GSO will present new methods for future selection problems

**Table 2:** Packet_delivery_ratio for slow heat-SA-GSO.

Packet rate (pkt/second)	SA-GSO	Slow heat-SA-GSO
2	0.8008	0.8229
4	0.7952	0.8159
6	0.7832	0.8025
8	0.7352	0.7509
10	0.6899	0.7102

**Table 3:** Average_delay in second for slow heat-SA-GSO.

Packet rate (pkt/second)	SA-GSO	Slow heat-SA-GSO
2	0.6819	0.6665
4	0.7002	0.6728
6	0.7377	0.7106
8	0.7581	0.7427
10	0.781	0.7566

**Table 4:** Average_number_of_hops for slow heat-SA-GSO.

Packet rate (pkt/second)	SA-GSO	Slow heat-SA-GSO
2	3.7	3.6
4	3.6	3.5
6	3.5	3.4
8	4.2	4
10	4.2	4

## Data Availability

The data used to support the findings of this study are included within the article.

## References

[1] Toutouh J., García-Nieto J., Alba E. Optimal configuration of OLSR routing protocol for VANETs by means of Differential Evolution.

[2] Xu S., Guo P., Xu B., Zhou H. (2013). QoS evaluation of VANET routing protocols. *Journal of Networks*.

[3] Mchergui A., Moulahi T., Alaya B., Nasri S. (2017). A survey and comparative study of QoS aware broadcasting techniques in VANET. *Telecommunication Systems*.

[4] Gupta K., Kohli A. P. S. (2016). Optimizing OLSR protocol for VANET. *Asian Journal of Technology and Management Research (AJTMR)*.

[5] Cui Y. Application of simulated annealing particle swarm optimization algorithm in power coal blending optimization.

[6] Khairi D., Berqia A. (2015). Survey on QoS and security in vehicular ad hoc networks. *International Journal*.

[7] Jothi K. R., Jeyakumar A. E. (2015). Optimization and quality-of-service protocols in VANETs: a review. *Artificial Intelligence and Evolutionary Algorithms in Engineering Systems*.

[8] Toutouh J., García-Nieto J., Alba E. (2012). Intelligent OLSR routing protocol optimization for VANETs. *IEEE Transactions on Vehicular Technology*.

[9] Pagadala P. K., Saravana Kumar N. M. (2021). Slow heat based simulated annealing and quality aware routing in vehicular ad hoc network. *Wireless Personal Communications*.

[10] Shaikh F. I., Hingoliwala H. A. Path planning based QoS routing in VANET.

[11] Zhao J., Wu Z., Wang Y., Ma X. (2019). Adaptive optimization of QoS constraint transmission capacity of VANET. *Vehicular Communications*.

[12] Bitam S., Mellouk A. (2013). Bee life-based multi constraints multicast routing optimization for vehicular ad hoc networks. *Journal of Network and Computer Applications*.

[13] Kasana R., Kumar S. A geographic routing algorithm based on Cat Swarm Optimization for vehicular ad-hoc networks.

[14] Kaur S., Aseri T. C., Rani S. QoS-Aware routing in vehicular ad hoc networks using ant Colony optimization and bee Colony optimization.

[15] Malathi A., Sreenath N. (2018). Improved shuffled frog-leaping algorithm based QoS constrained multicast routing for vanets. *Wireless Personal Communications*.

[16] Pagadala P., Saravana Kumar N. M. (2018). A Survey on Topology based Reactive Routing Protocols in Vanets. *Global Journal of Computer Science and Technology*.

[17] Fermani M., Rossit D. G., Toncovich A., Rossit D. A., Tohmé F., Mejía Delgadillo G. A simulated annealing algorithm for solving a routing problem in the context of municipal solid waste collection.

[18] Gautami R., Sedamkar R. R., Patil H. (2016). Application of hybrid metaheuristic algorithm for OLSR protocol optimization in VANET. *International Journal of Current Engineering and Technology*.

[19] Zhou Y., Zhou G., Zhang J. (2013). A hybrid glowworm swarm optimization algorithm for constrained engineering design problems. *Applied Mathematics & Information Sciences*.

[20] Sadati N., Zamani M., Mahdavian H. R. F. Hybrid particle swarm-based-simulated annealing optimization techniques.

[21] Urquiza-Aguiar L., Almeida D., Tripp-Barba C., Aguilar Igartua M. Heuristic methods in geographical routing protocols for VANETs.

[22] Urquiza-Aguiar L., Tripp-Barba C., Aguilar Igartua M. (2016). A geographical heuristic routing protocol for VANETs. *Sensors*.

[23] Liu J., Zhou Y., Huang K., Ouyang Z., Wang Y. (2011). A glowworm swarm optimization algorithm based on definite updating search domains. *Journal of Computational Information Systems*.

[24] Velleda Gonzales G., Domingues dos Santos E., Ramos Emmendorfer L., André Isoldi L., Oliveira Rocha L. A., Da Silva Diaz Estrada E. (2015). A comparative study of simulated annealing with different cooling schedules for geometric optimization of a heat transfer problem according to constructal design. *Scientia Plena*.

[25] Zhang J. (2013). Simulated Annealing: In Mathematical Global Optimization Computation, Hybrid with Local or Global Search, and Practical Applications in Crystallography and Molecular Modelling. https://arxiv.org/abs/1308.6220.

[26] Yarinezhad R., Sarabi A. (2019). A New Routing Algorithm for Vehicular Ad-Hoc Networks Based on Glowworm Swarm Optimization Algorithm. *Journal of AI and Data Mining*.

[27] Husnain G., Anwar S. (2022). An intelligent probabilistic whale optimization algorithm (i-WOA) for clustering in vehicular ad hoc networks. *International Journal of Wireless Information Networks*.

[28] Azzoug Y., Boukra A. (2021). Bio-inspired VANET routing optimization: an overview: a taxonomy of notable VANET routing problems, overview, advancement state, and future perspective under the bio-inspired optimization approaches. *Artificial Intelligence Review*.

[29] Saravana Kumar N. M., Pagadala P. K., Vijayakumar V., Kavinya A. (2022). Multi objective glow swarm based situation and quality aware routing in VANET. *Wireless Personal Communications*.

[30] Ingber L. (1989). Very fast simulated re-annealing. *Mathematical and Computer Modelling*.

[31] Kirkpatrick S., Gelatt C. D., Vecchi M. P. (1983). Optimization by simulated annealing. *Science*.

[32] Kalaiselvi T., Nagaraja P., Abdul Basith Z. (2017). A review on glowworm swarm optimization. *International Journal of Information Technology (IJIT)*.

[33] Kumar R. R., Babu J. M., Saleh B. (2023). Experimental and analytical investigation on friction welding dissimilar joints for aerospace applications. *Ain Shams Engineering Journal*.

[34] Pb S., Afzal A., Afzal A. (2022). The influence of exhaust gas recirculation on the characteristics of compression ignition engines powered by tamanu methyl ester. *International Journal of Low Carbon Technologies*.

[35] Zhou Y., Luo Q., Liu J. (2014). Glowworm swarm optimization for dispatching system of public transit vehicles. *Neural Processing Letters*.

[36] Pagadala P. K., Saravana Kumar N. M. (2010). Routing Protocols in Vehicular Ad Hoc Networks: A Survey. *Global Journal of Engineering Science and Researches*.

